# Efficacy and safety evaluation of gilvetmab in dogs with melanoma and mast cell tumor

**DOI:** 10.1093/jvimsj/aalag098

**Published:** 2026-06-05

**Authors:** Esther Chon, Mohamad Morsey, Terry Katz, Kazumi Yamada, Dennis Bailey, Philip J Bergman, Holly Burr, Craig A Clifford, Heather Heeb, Christina Manley, Brenda Phillips, Gerald Post, David M Vail, Anthony Rusk, Matthew L Stock

**Affiliations:** Merck Animal Health, Rahway, NJ, United States; Merck Animal Health, Rahway, NJ, United States; Merck Animal Health, Rahway, NJ, United States; Merck Animal Health, Rahway, NJ, United States; Oradell Animal Hospital, Paramus, NJ, United States; VCA Katonah Bedford Veterinary Center, Bedford Hills, NY, United States; VCA Clinical Studies, Los Angeles, CA, United States; Las Vegas Veterinary Specialty Center, Las Vegas, NV, United States; BluePearl Pet Hospital Malvern, Malvern, PA, United States; BluePearl Veterinary Specialty Hospital in Overland Park, Overland Park, KS, United States; The Oncology Service at the LifeCentre, Leesburg, VA, United States; Veterinary Specialty Hospital - Sorrento Valley, San Diego, CA, United States; MedVet Norwalk, Norwalk, CT, United States; Department of Medical Sciences, School of Veterinary Medicine, Madison, WI, United States; Carbone Comprehensive Cancer Center, University of Wisconsin-Madison, Madison, WI, United States; ACI Biosciences, LLC, Chevy Chase, MD, United States; Merck Animal Health, Rahway, NJ, United States

**Keywords:** cancer, canine, immune checkpoint inhibitor, immunotherapy, monoclonal antibody, PD-1, Programmed cell death 1 receptor

## Abstract

**Background:**

Immune checkpoint inhibitors (ICIs) have transformed oncology in human medicine, providing clinical benefit in a broad spectrum of cancers. Widely available ICIs for dogs are lacking.

**Hypothesis/Objectives:**

Evaluate efficacy and safety of gilvetmab, a caninized anti-PD-1 monoclonal antibody.

**Animals:**

Fifty-one client-owned dogs were evaluated, 25 with stages II-III melanoma and 26 with stages I-III mast cell tumor (MCT). Fifteen dogs with stages III-V lymphoma were also evaluated.

**Methods:**

Multi-institutional, open-label study. Enrolled dogs were treated with gilvetmab IV at 6 mg/kg q28d or 10 mg/kg q14d; 8 dogs receiving the lower dosage underwent dose escalation with their owners’ consent. Safety was evaluated by physical examinations, laboratory testing, and clinical observations made by veterinarians or the dogs’ owners. Efficacy was assessed by objective response rate (ORR) and time to progression (TTP) using cRECIST v1.0 and lymphoma response criteria.

**Results:**

For melanoma, the ORR was 20% (95% confidence interval [CI], 7%-41%) and median TTP was 56 days. For MCT, the ORR was 46% (95% CI, 27%-67%) and median TTP was not reached. No objective responses were observed in dogs with lymphoma. Serious adverse events of anaphylaxis, hypotension, or tumor hemorrhage occurred in 3 dogs (3/51, 5.9%). Tumor enlargement before regression, consistent with possible pseudoprogression, was observed in 2 dogs with melanoma.

**Conclusions and clinical importance:**

Gilvetmab has a reasonable expectation of efficacy and an acceptable preliminary safety profile in dogs with MCT stages I-III and melanoma stages II and III.

## Introduction

Immune checkpoint inhibitors (ICIs) have revolutionized the therapeutic landscape for numerous cancers in people.^1–7^ Immune checkpoints (ICs) are inhibitory immunoreceptors that act as gatekeepers of the immune response. A hallmark of cancer is immune evasion, and upregulation of ICs is one mechanism by which cancer cells exploit the body’s natural negative feedback mechanisms to co-opt immune suppression.^2^^,^^8^^,^^9^ Inhibiting the suppressive functions of ICs unleashes the antitumor response of immune cells.^1^^,^^4^^,^^5^

Many ICs have been investigated, including programmed cell death 1 (PD-1).^2^^,^^9^ It is a transmembrane protein expressed on the surface of T cells that binds to the coinhibitory molecules programmed cell death ligand 1 (PD-L1) and ligand 2 (PD-L2). These ligands are expressed on antigen-presenting cells among other cell types and are critical to maintaining self-tolerance by normal immune cells.^10^^,^^11^ Cancer cells also can express PD-L1/L2, which binds to PD-1 to enable its negative T-cell modulatory effects and thereby diminish T-cell antitumor efficacy.^11–13^ Immune checkpoint inhibition can be achieved by preventing ligand–receptor engagement using blocking antibodies.

Immune checkpoint inhibitors have resulted in increased patient survival in many cancer types including melanoma and non-small cell lung cancer.^6^^,^^9^^,^^14^^,^^15^ More than 50 ICIs have been approved by the Food and Drug Administration (FDA) for treatment of more than 19 types of cancer in humans.^16^^,^^17^ More ICIs and the ICs they target continue to be investigated.^18^^,^^19^

**Figure 1:**
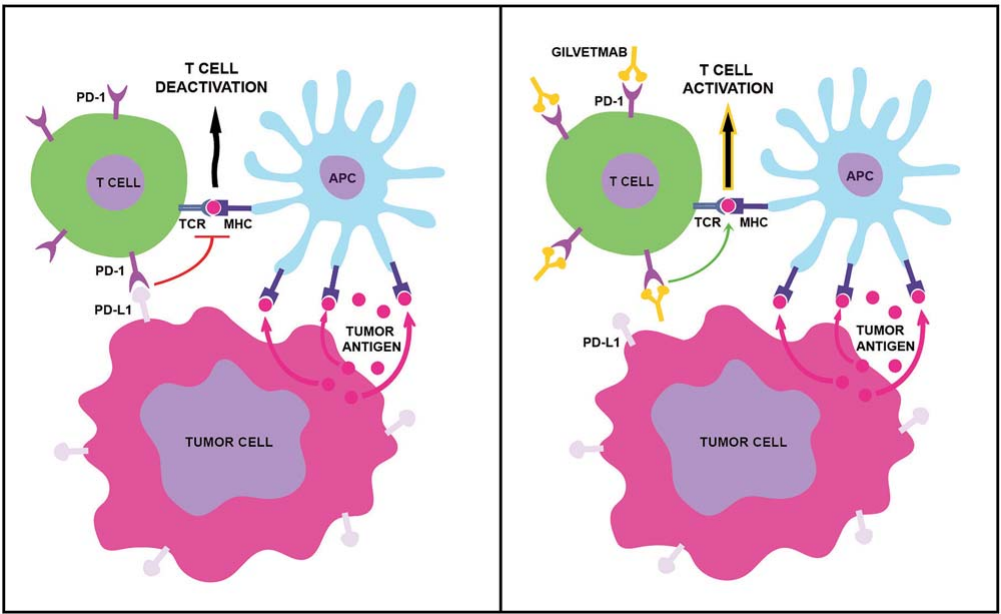
Tumor immune evasion via receptor-ligand engagement of the PD-1/PD-L1 pathway can be blocked with gilvetmab. The binding of PD-L1 on tumor cells to PD-1 on T cells affects many signaling pathways, including suppression of TCR/MHC engagement and subsequent signal transduction (left panel). Gilvetmab, a caninized PD-1 monoclonal antibody, blocks the PD-1/PD-L1 pathway, allowing for T cell activation (right panel). Abbreviations: MHC = major histocompatibility complex; PD-1 = programmed cell death receptor 1; PD-L1 = programmed cell death ligand 1; TCR = T cell receptor.

In dogs, a few ICIs have been investigated, including both anti-PD-1 and anti-PD-L1 antibodies.^20–26^ Despite measurable efficacy in tumor-bearing dogs,^21^^,^^22^^,^^25^ none of these ICIs are currently widely available. Gilvetmab is a caninized anti-PD-1 monoclonal antibody (Figure 1). Previous preclinical work has shown gilvetmab’s specificity and high affinity for canine PD-1, its pharmacological activity in blocking the interaction between PD-1 and its ligand PD-L1, and PD-1 target engagement on cluster of differentiation 4 (CD4) and cluster of differentiation 8 (CD8) T cells in healthy beagle dogs receiving gilvetmab IV.^27^

We conducted an open-label, multicenter study of this novel PD-1 inhibitor to identify a reasonable expectation of efficacy in dogs with measurable melanoma, mast cell tumor (MCT), and lymphoma. Melanoma was selected because of its high anti-PD-1 response rates in people^28–33^ as well as molecular and behavioral similarities between canine and human melanomas,^34–39^ whereas MCT and lymphoma were selected because of their high incidence among cancers in dogs.^40–42^ Our objectives were to evaluate gilvetmab’s efficacy and safety in cancer-bearing dogs. The study supported the conditional licensure of gilvetmab by the United States Department of Agriculture (USDA) for the treatment of dogs with melanoma and MCT. A USDA conditional license was not granted for gilvetmab to treat lymphoma because of a lack of reasonable expectation of efficacy as a monotherapy in this tumor type. Consequently, we focused on melanoma and MCT, and detailed information regarding lymphoma is not presented.

## Materials and methods

### Study overview

Ours was a multi-institutional, open-label, single-arm, prospective study. The study was conducted following Good Clinical Practice in accordance with USDA Veterinary Science Memorandum 800.301 and was approved by the Animal Care and Use Committees of Animal Clinical Investigation and University of Wisconsin-Madison. Dogs were recruited by investigators at hospital study sites.

### Patient eligibility

Dogs were screened between 7 and 0 days before beginning treatment. Before screening, written informed consent for participation was obtained from the dog’s owner or legal representative. At screening, performed assessments and collected information included patient breed, weight, age, sex/neuter status, medical history, physical examination findings, including measurement of lesion(s), Veterinary Cooperative Oncology Group—Common Terminology Criteria for Adverse Events (VCOG-CTCAE) v1.1 performance score evaluation,^43^ laboratory evaluations (CBC, serum biochemistry panel, and urinalysis), cytology or histopathology if a diagnosis of cancer had not been made before screening, 3-view thoracic radiographs (required for melanoma; optional for MCT), and abdominal ultrasound (required for MCT; optional for melanoma).

Inclusion criteria included signed informed consent before screening, client-owned dog ≥ 1 year of age, body weight ≥ 3 kg, cytologic or histopathologic diagnosis of melanoma or MCT of stages I-III for melanoma and stages I-III and substage a for MCT, ≥ 1 measurable lesion with a longest diameter of ≥ 2 cm at baseline, lesion amenable to biopsy or fine needle aspiration, and a VCOG Performance Score of 0 or 1 as defined by VCOG-CTCAE v1.1.^43^ Prior short-acting glucocorticoid treatment, chemotherapy, radiation therapy, or small molecule inhibitor therapy was acceptable after a 21-day washout period. Prior immunotherapy or targeted monoclonal antibody treatment was acceptable after a 42-day washout period. Prior surgery to remove masses was acceptable if measurable disease remained to meet inclusion criteria and was performed ≥ 14 days before enrollment. Prior use of nonsteroidal anti-inflammatory drugs was acceptable and allowed to continue during the study, if the dog had been receiving it for at least 30 days before enrollment. Any other concomitant medications that were not targeted for the treatment of cancer were acceptable. Dogs with multicentric lymphoma included stages I-V without pulmonary or gastrointestinal involvement, substage a, naïve or first relapse, and of B-cell, T-cell, or unknown immunophenotype.

Exclusion criteria included the presence of any underlying disease that in the opinion of the investigator would affect the study objectives or overall patient safety, substage b MCT, stage IV MCT or melanoma, reproductive status of pregnant or lactating or likely to become pregnant, participation in another study, and lack of availability for the entire study duration. During the study, dogs were not permitted to receive other concomitant medications targeted at the treatment of cancer, including holistic therapy, corticosteroids, immunotherapy, radiation therapy, small molecule inhibitors, surgery, and other investigational treatments.

### Trial design

Gilvetmab, a sterile liquid in single-use vials at a concentration of 20 mg/mL, was stored at refrigerator temperatures (2-8°C [35-46°F]). Dogs were premedicated with IM or PO with diphenhydramine at a dosage of approximately 2 mg/kg within 30 min or 4 h of treatment, respectively. Gilvetmab, undiluted, was administered IV over 30 min.

The initial evaluated gilvetmab dosage was 6 mg/kg q28d for up to 5 treatments. As the trial progressed, newly enrolled dogs were treated using an escalated dosage of 10 mg/kg IV q14d for up to 10 treatments to determine its impact on efficacy and safety. Dogs that were receiving or had received 6 mg/kg q28d for 5 treatments were allowed to continue at an escalated dosage of 10 mg/kg q14d for 5 treatments. The dosing regimens were based on laboratory dose determination studies evaluating serum concentrations and target engagement at various dosing regimens.^27^

Evaluations were performed according to dosage assignment (Table 1) and included medical history, physical examination findings including body weight and vital signs, laboratory evaluations, lesion measurement, VCOG performance score determination,^43^ quality-of-life (QoL) questionnaire,^44^ and imaging (thoracic radiographs, abdominal ultrasound, or both). Serum samples were collected immediately before and within 2 min after gilvetmab infusion to determine trough and peak serum concentrations, respectively (Method S1A). Peripheral blood mononuclear cells were collected immediately before each gilvetmab infusion to determine trough target engagement at 2-4 weeks posttreatment depending on the treatment regimen (Method S1B).

**Table 1:** Summary of study events by dose and study day.

**Day**	**Dose 6 mg/kg q28 days**	**Dose escalation** ^**a**^	**Dose 10 mg/kg q14 days**
**−7 to 0 (Screening)**	IC; HX; PE/VPS; LAB; CXR^b^; AUS^c^; LM^d^; IEC	IC; HX; PE/VPS; LAB; CXR^b^; AUS^c^; LM^d^; IEC	IC; HX; PE/VPS; LAB; CXR^b^; AUS^c^; LM^d^; IEC
**0^e^ (Enrollment)**	PE/VPS/QoL; LM; TX^f^	PE/VPS/QoL; LM; TX^f^	PE/VPS/QoL; LM; TX^f^
**14^e^**	PE/VPS/QoL; LAB	PE/VPS/QoL; LAB	PE/VPS/QoL; LAB; TX^f^
**28^e^**	PE/VPS/QoL; LAB; LMRE; TX^f^	PE/VPS/QoL; LAB; LMRE; TX^f^	PE/VPS/QoL; LAB; LMRE; TX^f^
**42^e^**			PE/VPS/QoL; LAB; TX^f^
**56^e^**	PE/VPS/QoL; LAB; CXR^b^; LMRE; TX^f^	PE/VPS/QoL; LAB; CXR^b^; LMRE; TX^f^	PE/VPS/QoL; LAB; CXR^b^; LMRE; TX^f^
**70^e^**			PE/VPS/QoL; LAB; TX^f^
**84^e^**	PE/VPS/QoL; LAB; CXR^b^; LMRE; TX^f^	PE/VPS/QoL; LAB; CXR^b^; LMRE; TX^f^	PE/VPS/QoL; CBC/chemistry/UA; CXR^b^; LMRE; TX^f^
**98^e^**			PE/VPS/QoL; CBC/chemistry/UA; TX^f^
**112^e^**	PE/VPS/QoL; LAB; LMRE; TX^f^	PE/VPS/QoL; LAB; LMRE; TX^f^	PE/VPS/QoL; LAB; LMRE; TX^f^
**126^e^**			PE/VPS/QoL; LAB; TX^f^
**140^e^**	PE/VPS/QoL; LAB; CXR^b^; AUS^c^ LMRE	IC; PE/VPS/QoL; LAB; CXR^b^; AUS^c^; LMRE; TX^f^	PE/VPS/QoL; LAB; CXR^b^; AUS^c^ LMRE
**154^e^**		PE/VPS/QoL;LAB; TX^f^	
**168^e^**		PE/VPS/QoL; LAB; LMRE; TX^f^	
**182^e^**		PE/VPS/QoL; LAB; TX^f^	
**196^e^**		PE/VPS/QoL; LAB; LMRE; TX^f^	
**210^e^**		PE/VPS/QoL; LAB; CXR^b^; AUS^c^; LMRE	

Abbreviations: AEs = adverse events; AUS = abdominal ultrasound; CXR = thoracic radiographs; HX = demographic and medical history; IC = informed consent; IEC = inclusion/exclusion criteria; LAB = laboratory tests including CBC = chemistry, and urinalysis; LM = lesion measurement; LMRE = lesion measurement and response evaluation; PE = physical exam; QoL = quality-of-life questionnaire; TX = gilvetmab treatment; VPS = VCOG Performance Score.

a Dose was given at 6 mg/kg q 28 days ×5, then not more than 8 weeks later, dose was escalated to 10 mg/kg q 14 days ×5.

b Thoracic radiographs was required for melanoma and lymphoma cases, optional for mast cell tumor cases.

c Abdominal ultrasound was required on mast cell tumor cases, optional for melanoma and lymphoma cases.

d Lesion measurement at screening visit was for inclusion/exclusion purposes only.

e AEs and concomitant medications were recorded at each visit, starting day 0.

f Vital signs (temperature, pulse, and respiration) recorded q2 hours ± 10 min for 6 h posttreatment. Posttreatment vital signs were included in AE assessment.

Dogs remained in the care of their owners and received their regular diets during the study. Dogs were removed from the study if one or more of the following occurred: progressive disease (PD) without owner or investigator interest in continuation, PD if performance score increased, unrelated medical or surgical condition development, owner noncompliance, an adverse event (AE) that required withdrawal, owner request, investigator judgment, or death. Necropsy was offered but not required for the study.

### Efficacy assessment

Objective response rate (ORR) was the primary endpoint for efficacy. The ORR was the proportion of all enrolled dogs that achieved a complete response (CR) or partial response (PR) based on the sum of the longest diameters of target lesions compared to baseline and as defined by cRECIST v1.0.^45^ Response in dogs with lymphoma was evaluated based on lymphoma response criteria.^46^ The best overall response was recorded between the start of treatment until study completion, early removal, or disease progression.

Time to progression (TTP), number of days from first treatment to time of PD, also was assessed. A minimum of one and a maximum of 5 target lesions were selected based on size and suitability for accurate repetitive measurements. All other measured lesions were considered nontarget lesions and were included for response evaluation.^45^ The longest diameter of each target lesion was measured using calipers, and the sum of target lesion diameters was calculated. An effort was made to have the same individual perform all lesion measurements for each dog. Assessment of distant PD was performed via imaging according to the study schedule (Table 1). If dogs continued to receive another cycle of treatment in the presence of equivocal PD, and progression was noted by the investigator at the next tumor assessment, PD was defined retroactively to the earlier date. Incisional biopsies were not performed to confirm progression.

### Safety assessment

Body temperature, pulse, and respiratory rate were monitored every 2 h for 6 h post-infusion, and AEs were recorded. Adverse events from observations made by a veterinarian (physical examination and laboratory evaluations) or reported by the dog’s owner were included. An AE was defined as any observation that was unfavorable, unintended, and occurred after the use of gilvetmab, whether or not the event was considered product-related, including worsening of an existing illness, a newly appearing disease, an accident, or a new finding in a clinical laboratory assessment. Adverse events were graded according to VCOG-CTCAE v1.1.^43^ Severity, causality (unknown, unlikely, possible, probable, or definite attribution of AE to gilvetmab), and outcome were recorded by the investigator. Adverse events were assessed throughout the study and were treated in accordance with standard veterinary medical practices. An adverse reaction (AR) was an AE that was determined to be possibly, probably, or definitely associated with the use of gilvetmab based on the investigator’s opinion of the potential contribution of gilvetmab weighed against the potential contribution of comorbidities (including the underlying cancer) to the event. Adverse reactions were defined as common if reported in > 10% of all dogs, at any VCOG grade and at any dose.

A serious adverse event (SAE) was defined as any medical occurrence that resulted in hospitalization longer than 24 h, that caused clinically relevant disability or morbidity, that was life threatening, or that resulted in death. The investigator made the final determination of whether or not an AE was categorized as serious.

Secondary safety evaluations included investigator assessment of a VCOG performance score^43^ and a QoL questionnaire^44^ completed by the owner.

### Statistical analyses

The sample size selected was based on the projected enrollment of a sufficient number of dogs with the specified tumor diagnosis to make a reasonable assessment of safety and efficacy. Based on results from health studies in humans evaluating anti-PD-1 monoclonal antibodies as monotherapies,^47–53^ an ORR of at least 20% was considered indicative of pharmacologic value.

All enrolled dogs were evaluated for safety; dogs available for at least 1 tumor response evaluation after the first dose were included for efficacy. Descriptive statistics were performed using SAS/STAT (Version 9.4). Confidence intervals for ORR were calculated by the Clopper–Pearson method. Because the study was not powered for statistical comparisons, none were performed.

## Results

### Patient population

Seventy-four client-owned dogs were screened at 9 study sites. Sixty-six dogs were enrolled, of which 25 had melanoma, 26 had MCT, and 15 had lymphoma. Eight dogs were not enrolled because of stage IV disease (*n* = 4), a different metastatic tumor type (*n* = 2), or presence of azotemia and hydronephrosis (*n* = 2). Signalment, baseline performance scores, and tumor stage were recorded (Table 2). One dog with melanoma initially categorized as stage III was retrospectively determined on serial thoracic radiograph review to have pulmonary metastasis and was enrolled as stage IV under a protocol deviation. For dogs with lymphoma, 8 had stage III, 4 had stage IV, and 3 had stage V lymphoma; 5 had relapsed lymphoma; 5 had previous chemotherapy treatments; and 9 had either T-cell or unknown immunophenotype.

**Table 2:** Baseline characteristics of dogs with melanoma and mast cell tumor.

	**Melanoma (*n* = 25)**	**MCT (*n* = 26)**
**Gender**		
** Male**	13 (52.0%)	13 (50.0%)
** Female**	12 (48.0%)	13 (50.0%)
**Age at screening (years)**		
** Mean**	12.1	9.1
** Std**	2.18	1.81
** Range**	6.4-16.2	6.4-13.1
** Median**	12	8.8
**Baseline weight (kg)**		
** Mean**	24.9	28.2
** Std**	14.6	12.8
** Range**	4.8-58.0	7.4-55.6
** Median**	25.2	29.1
**Breed**		
** Labrador Retriever**	5	4
** American Staffordshire Terrier**	1	5
** Cocker Spaniel**	3	1
** Golden Retriever**	1	2
** Pug**	2	1
** Boxer**	1	1
** Cane Corso**	2	0
** Scottish Terrier**	1	0
** Greater Swiss Mountain Dog**	0	1
** Greyhound**	0	1
** Miniature Pinscher**	0	1
** Miniature Schnauzer**	1	0
** Pomeranian**	0	1
** Shih Tzu**	1	0
** Mixed breed**	7	8
**Baseline performance score**		
** 0: Normal activity**	24 (96.0%)	25 (96.2%)
** 1: Mild lethargy; diminished activity**	1 (4.0%)	1 (3.8%)
**Tumor stage**		
** I**	0	14
** II**	7	3
** III**	17	9
** IV**	1^a^	Not applicable
** V**	Not applicable	Not applicable

a Stage IV dogs were excluded from the study with the exception of 1 dog that was not originally noted to have pulmonary metastasis at screening but was later retrospectively determined to have been present at the time of screening based on review of serial thoracic radiographs. This dog was therefore enrolled under a protocol deviation.

Most dogs (melanoma, *n* = 17, 68%; MCT, *n* = 14, 53.8%) had received prior treatment, and the most common method of diagnosis was cytology for both tumor types (Table 3). Of the 25 dogs with melanoma, 22 (88%) melanomas were oral, 2 (8%) were cutaneous, and 1 (4%) was digital. Of the 17 dogs with stage III melanoma, 8 (47%) had lymph node metastasis as the target lesion. Of the 26 dogs with MCT, all primary MCTs were cutaneous or subcutaneous. Two of these dogs with MCT (7.7%) had a lymph node metastasis as a target lesion.

**Table 3:** Prior therapies, method of diagnosis, and days to first gilvetmab treatment.

	**Melanoma (*n* = 25)**	**MCT (*n* = 26)**
**Prior therapy**	*n* (%)	
** No**	8 (32.0)	12 (46.2)
** Yes**	17 (68)	14 (53.8)
** Surgery only**	9 (36.0)	10 (38.5)
** Surgery + immunotherapy**	4 (16.0)^a^	0 (0)
** Surgery + chemotherapy ± prednisone**	0 (0)	2 (7.7)^b^
** Immunotherapy only**	2 (8.0)^a^	0 (0)
** Chemotherapy only**	2 (8.0)^c^	0 (0)
** Radiation + chemotherapy + prednisone**	0 (0)	1 (3.8)^d^
** Prednisone only**	0 (0)	1 (3.8)
**Diagnostic method**	*n* (%)	
** Cytology**	17 (68)	21 (80.8)
** Biopsy**	2 (8)	3 (11.5)
** Based on prior testing and current presentation**	6 (24)	2 (7.7)
**Days between prior therapy and gilvetmab**		
** Mean**	107.9	28.2
** Range**	20-240	7.4-55.6
** Median**	67.5	29.1

a Immunotherapy consisted of a canine melanoma vaccine.

b Chemotherapy consisted of vinblastine only or vinblastine and toceranib phosphate.

c Chemotherapy consisted of lomustine or cyclophosphamide.

d Chemotherapy consisted of vinblastine and toceranib phosphate.

### Treatment administration

There were 2 main dosing groups: 6 mg/kg q28d (melanoma, *n* = 14; MCT, *n* = 8) and 10 mg/kg q14d (melanoma, *n* = 10; MCT, *n* = 11). Twenty-one dogs (41.2%, melanoma, 6/25; MCT, 15/26) completed the study. The most common reason for study discontinuation was disease progression, followed by AE or owner request (Table 4). Most (14/15) dogs with lymphoma received 6 mg/kg q28d; the median number of treatments was 1 (range 1-10); and the most common reason for study discontinuation was PD (*n* = 11).

**Table 4:** Study completion and reasons for study discontinuation.

	**Melanoma (*n* = 25)**	**MCT (*n* = 26)**
**Completed the study**		
** Yes, *n* (%)**	6 (24.0%)	15 (57.7%)
** No, *n* (%)**	19 (76.0%)	11 (42.3%)
**Reason for discontinuation^a^**		
** Disease progression**	12	8
** Unrelated medical/surgical condition**	1	0
** Owner noncompliance**	2	0
** Adverse event**	4	6
** Owner request**	4	1
** Death**	0	0
** Other reason**	1	0

a Some dogs had more than one reason for discontinuation.

The median number of treatments was 4 (range, 1-10) and 5 (range, 1-10) for dogs with melanoma and MCT, respectively. The median number of treatments in dogs treated with 6 mg/kg q28d was 2.5 (range, 1-5; *n* = 14) and 4 (range, 1-5; *n* = 8) for dogs with melanoma and MCT, respectively. Dogs that completed the planned 5 treatments at 6 mg/kg q28d were 4/14 and 4/8 for melanoma and MCT, respectively, but 1 dog with melanoma was removed from the study because of PD before the final study visit. The median number of treatments at 10 mg/kg q14d was 4.5 (range, 1-10; *n* = 10) and 5 (range, 1-10; *n* = 11) in dogs with melanoma and MCT, respectively. Dogs that completed the planned 10 treatments at 10 mg/kg q14d were 3/10 and 4/11 for melanoma and MCT, respectively, but 1 dog with melanoma was removed from the study at the owner’s request (difficulty traveling to study site) before the final study visit. Eight dogs (melanoma, *n* = 1; MCT, *n* = 7) that had received gilvetmab 6 mg/kg q28d for 5 treatments were allowed to continue at an escalated dosage of 10 mg/kg q14d for 5 additional treatments. A median of 28.5 days (range, 18-56 days) elapsed between the different dosages, and all 8 dogs received a total of 10 treatments. Only 1 dog with lymphoma continued at an escalated dosage to receive a total of 10 treatments.

Gilvetmab was used concurrently with other medications, including antipruritics, antibiotics, antiemetics, anti-inflammatory products, and anesthetics (Table S2). No ARs were observed from the concurrent use of gilvetmab with other medications.

### Efficacy

**Figure 2:**
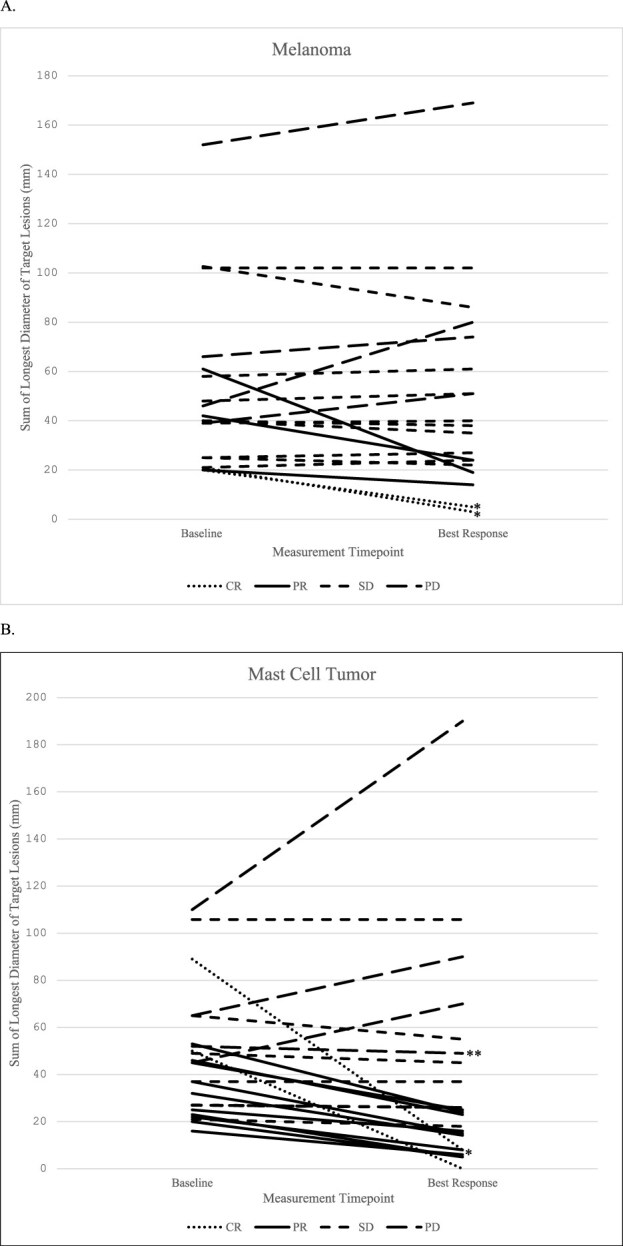
Change in sum of longest diameter of target lesions (mm) from baseline to best response in dogs with melanoma A and mast cell tumor B. The smallest sum of longest diameters was used if there were multiple visits with the same response. Three dogs (2 with melanoma and 1 with mast cell tumor) with progressive disease are not represented due to lack of follow-up measurements. Abbreviations: CR = complete response; PD = progressive disease; PR = partial response; SD = stable disease.

For all dogs, the ORR was 33% (17/51). The ORR for dogs with melanoma and MCT were 20% (5/25) and 46% (12/26), respectively (Tables 5 and S3).^54^ If dogs with stable disease (SD) were included, the clinical benefit (CR + PR + SD) was 66.7% (34/51). For both melanoma and MCT and for both dosages, objective responses (ORs) were observed and median days to response were similar (Table 6). Objective responses were observed for all MCT stages (I-III) and for melanoma stage III (Table 7; Figure 2).^54^ Stable disease was observed in 40% (10/25) and 27% (7/26) of dogs with melanoma and MCT, respectively. Two dogs with melanoma and 1 dog with MCT were not available for evaluation after the first dose, but these 3 dogs were defined conservatively as treatment failures and included in the denominator for ORR calculation. No ORs were observed for any lymphoma cases (ORR = 0%).

**Table 5:** Responses of dogs with melanoma or MCT.

**Response**	**Melanoma (*n* = 25)**	**MCT (*n* = 26)**
**CR (*n*, %)**	2 (8%)	2 (8%)
**PR (*n*, %)**	3 (12%)	10 (38%)
**SD (*n*, %)**	10 (40%)	7 (27%)
**PD (*n*, %)**	8 (32%)	6 (23%)
**NE (*n*, %)**	2 (8%)	1 (4%)
**Objective response (CR + PR)**	5	12
**ORR (%, 95% CI^a^)**	20% (7%-41%)	46% (27%-67%)

Abbreviations: CR = complete response; NE = not evaluable; ORR = objective response rate; PD = progressive disease; PR = partial response; SD = stable disease.

a Calculated by Clopper–Pearson method, which ignores factors such as cancer stage, breed, site, etc. For this reason, it is likely that the width of the CI is underestimated.

**Table 6:** ORs of dogs with melanoma or MCT by dose.

	**Melanoma (*n* = 25)**				**MCT (*n* = 26)**			
**Dose**	**# of subjects**	**# with OR (%)**	**Median days to OR (average, range)**	**Duration of Response (days)**	**# of subjects**	**# with OR (%)**	**Median days to OR (average, range)**	**Duration of response (days)**
**6 mg/kg q28 days**	15^a^	2^a^ (13.3%)	55 (55, 26-84)	86, 126+^b^	15^a^	7^a^ (46.7%)	56 (62.1, 28-155)	55+^b^, 56, 84, 84, 111, 112+^b^, 126
**10 mg/kg q14 days**	10	3 (30.0%)	58 (66, 56-58)	42, 55, 84+^b^	11	5 (45.5%)	56 (52, 27-91)	27, 49+^b^, 83+^b^, 83+^b^, 113+^b^

Abbreviations: MCT = mast cell tumor; ORs = objective responses.

a Dogs that experienced an OR (partial or complete response) are listed at the dose during which it was first observed.

b Still had an OR at end of study

**Table 7:** ORs of dogs with melanoma or MCT by stage.

	**Melanoma (*n* = 25)**			**MCT (*n* = 26)**		
**Stage**	**# of subjects**	**# with OR**	**(%)**	**# of subjects**	**# with OR**	**(%)**
**I**	0	0	0.0	14	7	50.0
**II**	7	0	0.0	3	1	33.3
**III**	17	5	29.4	9	4	44.4
**IV**	1^a^	0	0.0	Not applicable	Not applicable	0.0

Abbreviations: MCT = mast cell tumor; ORs = objective responses.

a Stage IV dogs were excluded from the study with the exception of 1 dog that was not originally noted to have pulmonary metastasis at screening but was later retrospectively determined to have been present at the time of screening based on review of serial thoracic radiographs. This dog was therefore enrolled under a protocol deviation.

**Figure 3:**
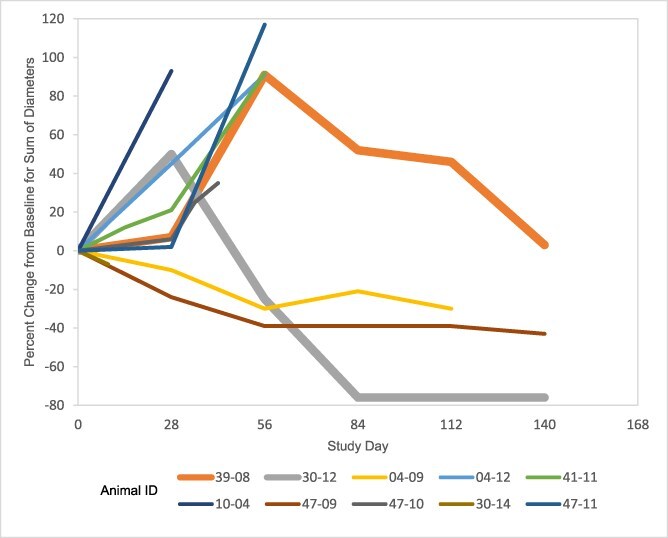
Responses of 10 dogs with melanoma receiving gilvetmab 10 mg/kg q 14 days. Initial tumor growth followed by regression for dogs 39-08 and 30-12 are depicted with thick lines to demonstrate possible pseudoprogression.

Two dogs with melanoma receiving gilvetmab at 10 mg/kg q14d initially were deemed to have PD but subsequently were noted to have tumor regression (Figure 3). In one of these dogs, a substantial increase (50%) in target lesion size was observed after 2 doses of gilvetmab (at day 28), followed by continuous decreases in the size of target lesions and ultimately CR at the day 84 visit that was maintained on day 112 before PD was noted in a nontarget lesion during an unscheduled visit on day 126. In the other dog, a 92% increase in the size of the target lesions occurred after 4 doses of gilvetmab (day 56) before a subsequent and continuous size decrease to 4% the baseline lesion measurement occurred by at the end of the trial (day 140). This patient was deemed to have SD as its best response. Both dogs continued to receive gilvetmab in the presence of initial tumor enlargement, and both dogs received all 10 scheduled doses.

The median TTP was 56 days for dogs with melanoma and not reached for dogs with MCT. At the end of the study, 14% of dogs with melanoma and 39% of dogs with MCT were progression-free.

### Safety

Dogs with melanoma and MCT remained on study for a median of 66 days (mean, 82.9 days; range, 10-211 days) and 140 days (mean, 121.8 days; range, 0-210 days), respectively. Common ARs in dogs with both tumor types were lethargy, fatigue, or poor general performance (15/51, 29.4%), decreased appetite (9/51, 17.6%), vomiting (7/51, 13.7%), weight loss (6/51, 11.88%), and increased alkaline phosphatase activity (6/51, 11.8%, Tables 8 and 9).^54^ Common ARs reported for melanoma were lethargy, fatigue, or poor general performance (6/25, 24%), decreased appetite (4/25, 16%), vomiting (4/25, 16%), and weight loss (4/25, 16%, Table 8).^54^ Common ARs reported for MCT were lethargy, fatigue, or poor general performance (9/26, 34.6%), decreased appetite (5/26, 19.2%), increased alkaline phosphatase activity (4/26, 15.4%), increased alanine aminotransferase activity (3/26, 11.5%), diarrhea (3/26, 11.5%), and vomiting (3/26, 11.5%; Table 9).^54^

**Table 8:** Adverse reactions (ARs) reported in dogs with melanoma.

**Adverse reaction** ^**a**^	**Any grade** ^**b**^ **AR (%)**	**Grades** ^**b**^ **3-5 (severe to life threatening) AR (%)**
**Lethargy/fatigue/general performance**	6 (24.0)	0 (0.0)
**Reduced appetite^c^**	4 (16.0)	0 (0.0)
**Vomiting**	4 (16.0)	0 (0.0)
**Weight loss**	4 (16.0)	0 (0.0)
**Alkaline phosphatase**	2 (8.0)	0 (0.0)
**Anemia^c^**	2 (8.0)	0 (0.0)
**Nausea/ptyalism**	2 (8.0)	0 (0.0)
**Proteinuria**	2 (8.0)	0 (0.0)
**Leukemia^c^**	1 (4.0)	1 (4.0)
**Monocytosis^c^**	1 (4.0)	1 (4.0)
**Muscle weakness, generalized or specific area**	1 (4.0)	1 (4.0)

Abbreviations: ARs = adverse reactions; VCOG-CTCAE = Veterinary Cooperative Oncology Group—Common Terminology Criteria for Adverse Events.

a ARs were described and graded following the VCOG-CTCAE v1.1.

b Only the worst grade of an adverse reaction in any given dog is included in the occurrence calculation.

c Reflects “Other (Specify, ___)” under the body system category per VCOG-CTCAE v1.1, using the most appropriate term from other coding dictionaries.

**Table 9:** ARs reported in dogs with MCT.

**Adverse reaction** ^**a**^	**Any grade** ^**b**^ **AR (%)**	**Grades** ^**b**^ **3-5 (severe to life threatening) AR (%)**
**Lethargy/fatigue/general performance**	9 (34.6)	0 (0.0)
**Reduced appetite^c^**	5 (19.2)	0 (0.0)
**ALP**	4 (15.4)	1 (3.8)
**ALT**	3 (11.5)	1 (3.8)
**Diarrhea**	3 (11.5)	0 (0.0)
**Vomiting**	3 (11.5)	0 (0.0)
**Muscle weakness, generalized or specific area**	2 (7.7)	1 (3.8)
**AST**	2 (7.7)	0 (0.0)
**Anemia^c^**	2 (7.7)	0 (0.0)
**Hypotension**	2 (7.7)	0 (0.0)
**Leukocytosis^c^**	2 (7.7)	0 (0.0)
**Nausea/ptyalism**	2 (7.7)	0 (0.0)
**Tremor**	2 (7.7)	0 (0.0)
**Weight loss**	2 (7.7)	0 (0.0)
**Anaphylaxis**	1 (3.8)	1 (3.8)
**Tumor hemorrhage^c^**	1 (3.8)	1 (3.8)

Abbreviations: ALP = alkaline phosphatase; ALT = alanine aminotransferase; ARs = adverse reactions; AST = aspartate aminotransferase; MCT = mast cell tumor; VCOG-CTCAE = Veterinary Cooperative Oncology Group—Common Terminology Criteria for Adverse Events.

a ARs were described and graded following the VCOG-CTCAE v1.1.

b Only the worst grade of an adverse reaction in any given dog is included in the occurrence calculation.

c Reflects “Other (Specify, ___)” under the body system category per VCOG-CTCAE v1.1, using the most appropriate term from other coding dictionaries.

Non-serious AEs considered possibly or probably related to gilvetmab were reported for 6 dogs. Five of these dogs (melanoma, *n* = 3; MCT, *n* = 2) vomited during or after infusion, and all dogs recovered after administration of antiemetic medication. One dog with MCT developed ulcerated skin lesions of the lip folds, vulva, and perianal region that were biopsy-confirmed as a delayed hypersensitivity event. The skin lesions resolved after treatment with corticosteroids. The dog remained on study, but the OR was not included in the analysis from the date after initiation of corticosteroid treatment because of its possible impact on efficacy.

Serious adverse events were observed in 7/51 (13.7%) of dogs with melanoma (*n* = 3) and MCT (*n* = 4). Proportions of SAEs appeared similar between both dosage rates, with SAEs in 4/30 (13.3%) and 3/29 (10.3%) for 6 and 10 mg/kg, respectively (denominators for both dosages included dogs that underwent dose escalation because those dogs received both dosages; Table 10).^54^ No SAEs were observed in the 8 dogs that received both dosages.

**Table 10:** Serious adverse events (SAEs).

**SAE**	***n***	**Tumor type (stage)**	**Gilvetmab dose**	**Gilvetmab attribution**	**Disease progression attribution**
**Dysphagia**	1	Melanoma (3a)	6 mg/kg q28 days	Unlikely	Probably
**Vomiting with anorexia**	1	Melanoma (3a)	6 mg/kg q28 days	Unlikely	Probably
**Lethargy/fatigue/general performance**	1	MCT (3a)	6 mg/kg q28 days	Unlikely	Probably
**Dyspnea**	1	Melanoma (2a)	10 mg/kg q14 days	Unlikely	Unlikely
**Tumor hemorrhage^a^**	1	MCT (1a)	10 mg/kg q14 days	Possibly	Probably
**Anaphylaxis**	1^b^	MCT (1a)	6 mg/kg q28 days	Definitely	Unlikely
**Hypotension**	1	MCT (1a)	10 mg/kg q14 days	Definitely	Unlikely

Abbreviation: MCT, mast cell tumor.

a Hemorrhage was subsequent to rupture of a cutaneous MCT.

b SAE reported on 2 occasions.

Of the 7 dogs that had SAEs, 4 were considered unlikely and 3 (3/51, 5.9%) either possibly (*n* = 1) or definitely (*n* = 2) related to gilvetmab. Anaphylaxis and hypotension were 2 SAEs considered definitely related to gilvetmab. Both of these occurred in dogs with MCT during gilvetmab infusion, and resolved with infusion cessation and supportive care (fluid therapy with or without additional diphenhydramine). Anaphylaxis occurred in one dog during the day 28 infusion at 6 mg/kg and again on day 35 when another infusion was attempted. This dog was withdrawn from the study as a consequence. Hypotension occurred in another dog during the day 14 infusion at 10 mg/kg. This dog was withdrawn from the study at the owner’s request 2 days later because of tumor ulceration and bleeding. The SAE considered possibly related to gilvetmab was tumor hemorrhage in a dog from self-trauma to its MCT that ruptured and subsequently bled 7 days after the first (and only) infusion at 10 mg/kg. This dog was removed from the study and euthanized at the owner’s request.

Laboratory abnormalities included increased liver enzyme activities in 2 melanoma and 9 MCT cases; and anemia, proteinuria, leukocytosis, leukemia, and monocytosis in 2 or fewer melanoma or MCT cases. Leukemia (characterized as a B cell chronic lymphocytic leukemia), monocytosis, increased alkaline phosphatase activity, and increased alanine aminotransferase activity were grade 3 in 1 case each; all other laboratory abnormalities were grades 1 or 2 (Tables 8 and 9).^54^

Of the 50 dogs that had at least one follow-up performance score, 42 dogs (42/50, 84%) maintained a performance score of 0 or 1 while on study. The other 8 dogs had an increase in performance score during the study (melanoma, *n* = 5; MCT, *n* = 3).

Throughout the study, dogs generally maintained a favorable QoL based on the statement “My pet enjoys life,” which was selected from the questionnaire^44^ to represent overall QoL status. For dogs with melanoma, 92% (22/24) of owners indicated either “agree” to “slightly agree” (20/24) or “neutral” (2/24) to this statement. For dogs with MCT, 100% (25/25) of owners answered either “agree” to “slightly agree” (22/25) or “neutral” (3/25) to this statement. Collectively, 96% (47/49) of dogs with melanoma or MCT maintained a favorable QoL based on their owners’ neutral, slight, or full agreement of the statement “My pet enjoys life.”

### Serum concentration and target engagement analyses

**Figure 4:**
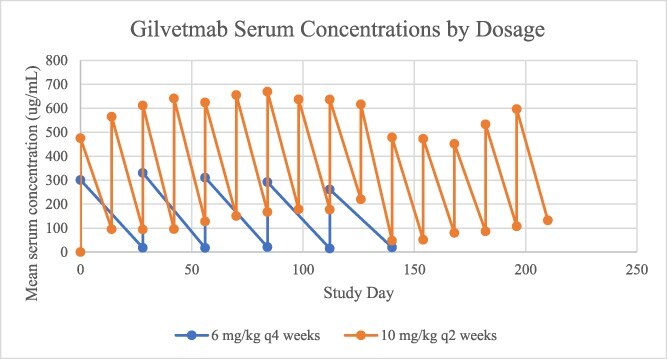
Serum gilvetmab concentrations immediately prior to and after gilvetmab infusion in dogs with melanoma, mast cell tumor, or lymphoma receiving gilvetmab 6 mg/kg q 28 days or 10 mg/kg q 14 days.

**Figure 5:**
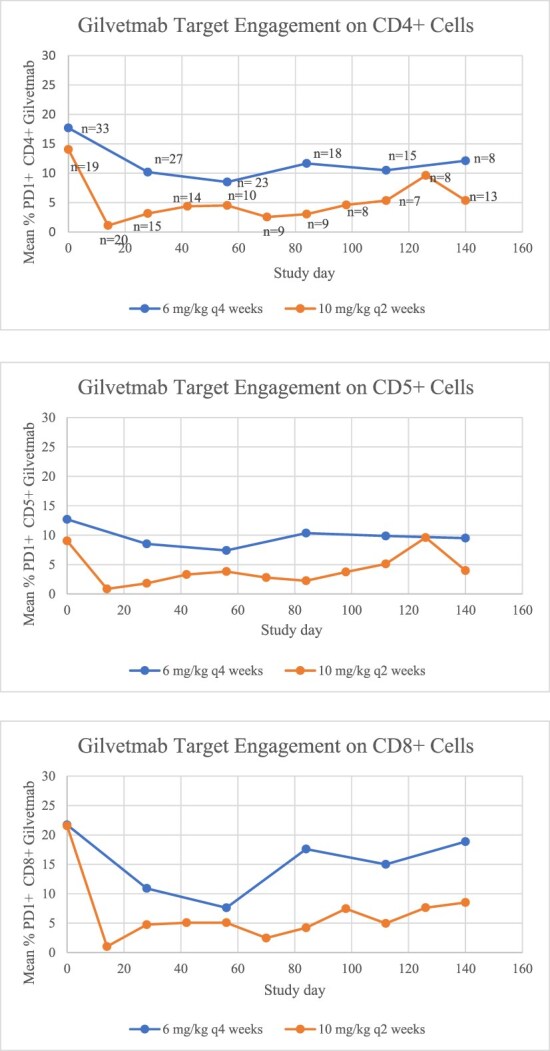
Target engagement in peripheral blood mononuclear cells obtained immediately prior to gilvetmab infusion in dogs with melanoma, mast cell tumor, or lymphoma receiving gilvetmab 6 mg/kg q 28 days or 10 mg/kg q 14 days. Target engagement was assessed by flow cytometric measurement of lymphocyte PD-1 receptors remaining available for ex vivo gilvetmab binding. Because this assay reflects receptor occupancy, higher target engagement is represented by lower available receptor percentages. Included are data points for which sample size was ≥ 5 patients. Sample sizes shown for the first panel apply to all panels. Abbreviation: PD-1 = programmed cell death receptor 1.

Trough and peak serum concentrations ranged from undetectable to 71.7 μg/mL, and 6.8 to 523.3 μg/mL, respectively, in dogs receiving 6 mg/kg q28d; and undetectable to 464.7 μg/mL, and 83.5 to 1054 μg/mL, respectively, in dogs receiving 10 mg/kg q14d (Figure 4). Five posttreatment samples were marked as undetectable, but the underlying reason was unclear. Gilvetmab binding to its receptors on T lymphocytes (CD4+, CD5+, and CD8+) occurred (Figure 5).

## Discussion

Gilvetmab is the first widely available and USDA conditionally licensed caninized anti-PD-1 monoclonal antibody in veterinary medicine. We demonstrated a reasonable expectation of efficacy and preliminary safety when gilvetmab was used as monotherapy in dogs with melanoma and MCT, with an ORR of 33% and a clinical benefit of 66.7% for all dogs, and an ORR of 20% (5/25) and 46% (12/26) for dogs with melanoma and MCT, respectively. Gilvetmab generally was well tolerated, with 2 infusion reactions that were self-limiting with supportive care. Favorable performance scores and QoL were maintained in most dogs (84% and 96%, respectively).

The observed ORRs in dogs receiving gilvetmab monotherapy align with those observed in people receiving anti-PD-1/PD-L1 monotherapy. According to a recent review, pan-cancer ORRs ranged from 0% to 38.8% in 31 cancer types and subtypes in over 10 000 people receiving anti-PD-1/PD-L1 monotherapy.^55^ Cancer is not commonly treated using monotherapy, and the combination of ICIs with other conventional treatments has demonstrated synergy manifested by improved outcomes.^29^^,^^56–58^ For example, chemotherapy, which can enhance tumor immunogenicity, can improve the effects of ICIs, resulting in enhanced survival time (ST) and ORR compared to either treatment alone. In humans with non-small cell lung cancer, the effects have been as notable as 64% ORR and a median ST not reached for ICI-chemotherapy combination as compared with 27% ORR and median ST of 24.8 months for ICI monotherapy.^59^^,^^60^ Such successes have led to several ICI-chemotherapy combination approvals in human medicine by the FDA and European Medicines Agency.^56^^,^^61^ Similar successes also have been demonstrated with other combinations, such as those combining ICI with surgery,^29^^,^^58^^,^^61^ radiation,^57^ and other treatments.^62–64^ With these demonstrable successes in people, it is feasible that dogs can also benefit from improved outcomes with ICI combination treatments. Trials evaluating combination treatments in veterinary medicine are currently under way.^65^^,^^66^ However, it has become increasingly apparent in people that several factors, such as timing of ICIs with any of the other combined treatment modalities and their doses, can impact outcomes.^29^^,^^67^ Therefore, there is a need to identify rational therapeutic combinations that will maximize efficacy and minimize toxicities.

Nine dogs with melanoma and MCT had ORs while receiving a lower dosage of gilvetmab (6 mg/kg q28d). The ORRs appeared similar between the lower and higher dosages, but there were not enough dogs to conclude similar efficacy between dosages. Therefore, larger prospective and controlled studies are needed to determine gilvetmab’s efficacy at a lower dosage.

Pseudoprogression is a phenomenon that is well documented in a subset of people receiving ICIs and presents as a transient increase in tumor size or the appearance of new lesions with subsequent regression.^68^ In people, it has a reported incidence of < 10%.^68^ Ideally, pseudoprogression is distinguished from true progression to avoid premature cessation of treatment. Currently, pseudoprogression in people is diagnosed using retrospective imaging analysis, but other methods (eg, liquid biopsy, lymphocyte assessment of biopsy samples) have been proposed to diagnose it in real time.^69^ Pseudoprogression in people can occur as early as within approximately 12 weeks of starting ICI treatment to beyond 12 weeks.^70^ To address the possibility of pseudoprogression in people, the original RECIST v1.1 criteria were modified to immune-RECIST to account for fluctuations in tumor size based on sequential tumor measurements within 4-8 weeks of when increased tumor size was first noted.^71–73^ Earlier studies have suspected pseudoprogression in 2 dogs receiving 2 different experimental anti-PD-1 antibodies. One dog had stage IV oral malignant melanoma with perceived PD based on enlarging pulmonary metastatic lesions 9 months after initiating treatment and later achieved CR at approximately 14 months.^74^ The other dog had salivary adenocarcinoma with perceived PD 6 weeks after initiating treatment with subsequent PR at 10 weeks that was maintained for 6 months until the dog was euthanized for an unrelated non-neoplastic issue.^25^ Two dogs in our study were deemed to have initial PD based on increased tumor size, with peak tumor increases occurring at days 28 and 56 followed by subsequent regression (Figure 3). The potential to determine subsequent regression was not built into our study design, and therefore response may be underrepresented in our study without applying immune-RECIST criteria^72^ as in people receiving ICIs. The clinical course in these dogs suggests that pseudoprogression may occur in dogs treated with gilvetmab. In dogs, no definitive methods are available to differentiate true progression from pseudoprogression, and mistaking one for the other can have potentially devastating effects (eg, premature euthanasia or discontinuation of effective treatment). Therefore, additional studies are warranted to determine if pseudoprogression occurs in dogs receiving ICIs. Until such methods are available, this phenomenon will remain a challenging aspect of clinical decision-making by veterinarians utilizing ICIs.

Although gilvetmab generally appeared well tolerated, 3 dogs with MCT experienced SAEs of anaphylaxis, hypotension, or skin hemorrhage that were deemed definitely or possibly attributable to gilvetmab. Anaphylaxis and hypotension occurred during gilvetmab infusion in 2 of these dogs (4%, 2/51) consistent with infusion reactions that may occur after monoclonal antibody administration as a result of IgE- or non-IgE-mediated mechanisms or secondary to cytokine release.^75^ This infusion reaction rate is similar to and falls within the range of 1%-6% of people receiving ICIs reported to experience infusion reactions. In people, fewer than 1% of such events are grade 3 or higher.^76–79^ In a third dog with MCT, skin hemorrhage from MCT rupture was deemed possibly related to gilvetmab and was noted 7 days after the first gilvetmab treatment at 10 mg/kg. The tumor may have ruptured because of continued enlargement from PD or pseudoprogression. This dog also was reported to have been self-traumatizing its MCT, which may have contributed to rupture, and underlying pruritus may have been the impetus for self-trauma. Although MCTs by their nature can induce pruritus, people receiving ICIs also have reported pruritus as an immune-related cutaneous AR or as a symptom of an immediate drug hypersensitivity reaction.^78^^,^^80^ Although SAEs were uncommon in our study population, they appeared with similar frequencies in both dosing regimens.

Autoimmune and autoinflammatory AEs related to immune activation, referred to as immune-related adverse events (irAEs),^81^ are a unique aspect of ICI toxicity in people, affecting more than half of people receiving ICIs and resulting in serious and potentially fatal consequences in some patients.^78^^,^^79^^,^^82^^,^^83^ Although serious irAEs were not apparent in our population of dogs receiving gilvetmab, the increased alanine aminotransferase activities observed in 5 dogs (all low grade except for a grade 3 increase in 1 dog) could reflect irAEs, but their non-pathognomonic nature and alternative plausible causes preclude definitive attribution as irAEs. The most common irAEs encountered in people include colitis, hepatotoxicity, skin rash, hyperthyroidism, and hypothyroidism,^83^ but the irAEs contributing to most fatalities are encephalitis, pneumonitis, colitis, and myocarditis.^84^ These irAEs most commonly occur within the first few weeks to months after ICI initiation, but they can occur at any time, including years after cessation of treatment.^84^^,^^85^ Moreover, timing of irAE onset can vary widely depending on the type of irAE and specific ICI used.^86^ A previous study reported suspected irAEs in 2 dogs that were receiving a different anti-PD-1 antibody. Sterile nodular panniculitis occurred in both dogs, and in one of these dogs, megaesophagus, myasthenia gravis, and hypothyroidism occurred simultaneously on day 455 after concurrent toceranib phosphate was initiated on day 385.^87^ Although we observed no definitive irAEs in our study population, dogs were not followed for the frequency of potential irAEs that could have occurred after conclusion of the study. Therefore, ICI-treated dogs that develop clinical signs inconsistent with the underlying cancer or the ARs identified in our study should be further evaluated for the possibility of irAEs.

When our study was conducted, dogs with multicentric lymphoma were also enrolled. However, all of these dogs with lymphoma did not achieve an OR when gilvetmab was given as monotherapy. Potential reasons for these results in these dogs with lymphoma include the facts that nearly half had stage IV or V lymphoma; one-third had relapsed lymphoma after having completed previous lymphoma therapy, and nearly half had either unknown or T-cell immunophenotype, all of which are predictive of aggressive behavior. Interestingly, ICIs have been effective in people with lymphoma,^88^ with some enjoying durable stable disease even without OR.^89^ In our study, one dog with lymphoma (stage IIIa, naïve, T-cell, and dose-escalated) that progressed after initial stable disease remained enrolled in the study for 219 days and received all 10 gilvetmab treatments because of continued normal activity performance score (0) despite slow progression that included waxing and waning of the size of lymph nodes. However, the most robust responses are seen in people with Hodgkin’s lymphoma,^88^ whereas lymphoma in dogs bears marked similarities with its less treatable and more common counterpart, non-Hodgkin’s lymphoma.^41^^,^^90^

Although formal pharmacokinetic analysis was not performed because sample collection did not occur beyond immediately posttreatment, trough and peak average serum concentrations appeared to increase with increased dosage. Target engagement immediately before treatment was observed in dogs treated with either regimen. However, it appears that target binding increased with the dosing regimen of 10 mg/kg q14d compared with the target engagement observed with a dosing regimen of 6 mg/kg q28d. Importantly, target engagement was evaluated at trough concentrations. Therefore, it is feasible that higher engagement could be noted at earlier time points, at either dose. Because numbers of responders were relatively small, definitive correlations between response and serum concentrations or target engagement cannot be made.

Our study had some limitations. Dogs with spontaneously occurring cancer of different stages and sites (eg, oral vs cutaneous melanoma) were evaluated to demonstrate reasonable expectation of efficacy and safety of gilvetmab. However, a larger population of dogs for each stage and site of cancer would have allowed for a more robust evaluation of gilvetmab’s efficacy in earlier as compared with more advanced stages of cancer and with different tumor locations. A study with increased numbers would be beneficial for evaluating gilvetmab in a larger heterogeneous population. Although according to cRECIST, calipers are an acceptable method of tumor measurement, future studies should include advanced imaging of tumors to corroborate caliper measurements in assessing response. Blinded and randomized studies would mitigate unintentional investigator bias.

We have demonstrated a reasonable expectation of efficacy and safety for gilvetmab use in dogs with melanoma and MCT. Efficacy and safety are currently being confirmed in an ongoing larger registration trial. Future considerations include identification of actionable predictive biomarkers, optimal patient selection, and effective patient monitoring to maximize drug efficacy and minimize toxicity. Combining gilvetmab with other treatment modalities would be expected to enhance the overall anticancer benefits of any single modality, and thus rational combination strategies should be investigated to maximize the benefits of combination therapy. The development of gilvetmab and demonstration of a reasonable expectation of efficacy in dogs will shift the paradigm of cancer therapy for our patients.

## Supplementary Material

S1-clean_aalag098

Table_S2-clean_aalag098

Table_S3-clean_aalag098

## Off-label antimicrobial declaration

Antimicrobials were administered to dogs in this study at the discretion of the overseeing clinician. Culture and sensitivity were not necessarily performed to inform and confirm on-label use in every dog.

## Institutional animal care and use committee or other approval declaration

Informed consent was obtained from the owners or legal custodians of all animals described in this study. This study was approved by the Animal Care and Use Committees of Animal Clinical Investigation, LLC and University of Wisconsin-Madison.

## Notes

Present addresses: Terry Katz is not currently affiliated with Merck Animal Health, Rahway, NJ, United States. Kazumi Yamada is not currently affiliated with Merck Animal Health, Rahway, NJ, United States. Gerald Post, OneHealth Company, Palo Alto, CA, United States. Philip J. Bergman, Veterinary and Cancer Immunotherapy Programs, Focused Ultrasound Foundation, Charlottesville, VA, United States.

## Abbreviations

AE: adverse event
AR: adverse reaction
CD4: cluster of differentiation 4
CD8: cluster of differentiation 8
CR: complete response
CTLA-4: cytotoxic T lymphocyte antigen 4
FDA: Food and Drug Administration
IC: immune checkpoint
ICI: immune checkpoint inhibitor
irAE: immune-related adverse event
MCT: mast cell tumor
OR: objective response
ORR: objective response rate
QoL: quality of life
PD: progressive disease
PD-1: programmed cell death 1
PD-L1: programmed cell death ligand 1
PD-L2: programmed cell death ligand 2
PR: partial response
SAE: serious adverse event
SD: stable disease
ST: survival time
TTP: time to progression
USDA: United States Department of Agriculture
VCOG-CTCAE: Veterinary Cooperative Oncology Group—Common Terminology Criteria for Adverse Events

## References

[1] Alturki NA . Review of the immune checkpoint inhibitors in the context of cancer treatment. J Clin Med. 2023;12:12. 10.3390/jcm12134301 PMC10342855 37445336

[2] He X, Xu C. Immune checkpoint signaling and cancer immunotherapy. Cell Res. 2020;30:660-669. 10.1038/s41422-020-0343-4 32467592 PMC7395714

[3] Johnson DB, Nebhan CA, Moslehi JJ, Balko JM. Immune-checkpoint inhibitors: long-term implications of toxicity. Nat Rev Clin Oncol. 2022;19:254-267. 10.1038/s41571-022-00600-w 35082367 PMC8790946

[4] Pardoll DM . The blockade of immune checkpoints in cancer immunotherapy. Nat Rev Cancer. 2012;12:252-264. 10.1038/nrc3239 22437870 PMC4856023

[5] Sharma P, Goswami S, Raychaudhuri D, et al. Immune checkpoint therapy-current perspectives and future directions. Cell. 2023;186:1652-1669. 10.1016/j.cell.2023.03.006 37059068

[6] Shiravand Y, Khodadadi F, Kashani SMA, et al. Immune checkpoint inhibitors in cancer therapy. Curr Oncol. 2022;29:3044-3060. 10.3390/curroncol29050247 35621637 PMC9139602

[7] Xu H, Yang Y, Yan Y, et al. Safety and efficacy of rechallenge with immune checkpoint inhibitors in advanced solid tumor: a systematic review and meta-analysis. Cancer Med. 2024;13:e70324. 10.1002/cam4.70324 39463070 PMC11513547

[8] Hanahan D . Hallmarks of cancer: new dimensions. Cancer Discov. 2022;12:31-46. 10.1158/2159-8290.CD-21-1059 35022204

[9] Seidel JA, Otsuka A, Kabashima K. Anti-PD-1 and anti-CTLA-4 therapies in cancer: mechanisms of action, efficacy, and limitations. Front Oncol. 2018;8:86. 10.3389/fonc.2018.00086 29644214 PMC5883082

[10] Ghosh C, Luong G, Sun Y. A snapshot of the PD-1/PD-L1 pathway. J Cancer. 2021;12:2735-2746. 10.7150/jca.57334 33854633 PMC8040720

[11] Lin X, Kang K, Chen P, et al. Regulatory mechanisms of PD-1/PD-L1 in cancers. Mol Cancer. 2024;23:108. 10.1186/s12943-024-02023-w 38762484 PMC11102195

[12] Dong H, Strome SE, Salomao DR, et al. Tumor-associated B7-H1 promotes T-cell apoptosis: a potential mechanism of immune evasion. Nat Med. 2002;8:793-800. 10.1038/nm730 12091876

[13] Han Y, Liu D, Li L. PD-1/PD-L1 pathway: current researches in cancer. Am J Cancer Res. 2020;10:727-742 32266087 PMC7136921

[14] Huang Q, Zheng Y, Gao Z, Yuan L, Sun Y, Chen H. Comparative efficacy and safety of PD-1/PD-L1 inhibitors for patients with solid tumors: a systematic review and Bayesian network meta-analysis. J Cancer. 2021;12:1133-1143. 10.7150/jca.49325 33442411 PMC7797652

[15] Ribas A, Puzanov I, Dummer R, et al. Pembrolizumab versus investigator-choice chemotherapy for ipilimumab-refractory melanoma (KEYNOTE-002): a randomised, controlled, phase 2 trial. Lancet Oncol. 2015;16:908-918. 10.1016/S1470-2045(15)00083-2 26115796 PMC9004487

[16] Robert C . A decade of immune-checkpoint inhibitors in cancer therapy. Nat Commun. 2020;11:3801. 10.1038/s41467-020-17670-y 32732879 PMC7393098

[17] Sharma P, Siddiqui BA, Anandhan S, et al. The next decade of immune checkpoint therapy. Cancer Discov. 2021;11:838-857. 10.1158/2159-8290.CD-20-1680 33811120

[18] Andrzejczak A, Karabon L. BTLA biology in cancer: from bench discoveries to clinical potentials. Biomark Res. 2024;12:8. 10.1186/s40364-024-00556-2 38233898 PMC10795259

[19] Joller N, Anderson AC, Kuchroo VK. LAG-3, TIM-3, and TIGIT: distinct functions in immune regulation. Immunity. 2024;57:206-222. 10.1016/j.immuni.2024.01.010 38354701 PMC10919259

[20] Choi JW, Withers SS, Chang H, et al. Development of canine PD-1/PD-L1 specific monoclonal antibodies and amplification of canine T cell function. PLoS One. 2020;15:e0235518. 10.1371/journal.pone.0235518 32614928 PMC7332054

[21] Igase M, Nemoto Y, Itamoto K, et al. A pilot clinical study of the therapeutic antibody against canine PD-1 for advanced spontaneous cancers in dogs. Sci Rep. 2020;10:18311. 10.1038/s41598-020-75533-4 33110170 PMC7591904

[22] Maekawa N, Konnai S, Takagi S, et al. A canine chimeric monoclonal antibody targeting PD-L1 and its clinical efficacy in canine oral malignant melanoma or undifferentiated sarcoma. Sci Rep. 2017;7:8951. 10.1038/s41598-017-09444-2 28827658 PMC5567082

[23] Minoli L, Licenziato L, Kocikowski M, et al. Development of monoclonal antibodies targeting canine PD-L1 and PD-1 and their clinical relevance in canine apocrine gland anal sac adenocarcinoma. Cancers (Basel). 2022;14:14. 10.3390/cancers14246188 PMC9777308 36551672

[24] Nemoto Y, Shosu K, Okuda M, Noguchi S, Mizuno T. Development and characterization of monoclonal antibodies against canine PD-1 and PD-L1. Vet Immunol Immunopathol. 2018;198:19-25. 10.1016/j.vetimm.2018.02.007 29571514

[25] Xu S, Xie J, Wang S, et al. Reversing stage III oral adenocarcinoma in a dog treated with anti-canine PD-1 therapeutic antibody: a case report. Front Vet Sci. 2023;10:1144869. 10.3389/fvets.2023.1144869 37252387 PMC10219605

[26] Yoshimoto S, Chester N, Xiong A, et al. Development and pharmacokinetic assessment of a fully canine anti-PD-1 monoclonal antibody for comparative translational research in dogs with spontaneous tumors. MAbs. 2023;15:2287250. 10.1080/19420862.2023.2287250 38047502 PMC10793675

[27] 2021 ACVIM Forum Research Abstract Program . 2021 ACVIM Forum Research Abstract Program. J Vet Intern Med. 2021;35:2943-3079. 10.1111/jvim.16220 34351021 PMC8692203

[28] Khushalani NI, Vassallo M, Goldberg JD, et al. Phase II clinical and immune correlate study of adjuvant nivolumab plus ipilimumab for high-risk resected melanoma. J Immunother Cancer. 2022;10:10. 10.1136/jitc-2022-005684 PMC9717375 36450385

[29] Patel SP, Othus M, Chen Y, et al. Neoadjuvant-adjuvant or adjuvant-only pembrolizumab in advanced melanoma. N Engl J Med. 2023;388:813-823. 10.1056/NEJMoa2211437 36856617 PMC10410527

[30] Rizzetto G, De Simoni E, Molinelli E, et al. Efficacy of pembrolizumab in advanced melanoma: a narrative review. Int J Mol Sci. 2023;24:12383. 10.3390/ijms241512383 PMC10419154 37569757

[31] Robert C, Schachter J, Long GV, et al. Pembrolizumab versus ipilimumab in advanced melanoma. N Engl J Med. 2015;372:2521-2532. 10.1056/NEJMoa1503093 25891173

[32] Schachter J, Ribas A, Long GV, et al. Pembrolizumab versus ipilimumab for advanced melanoma: final overall survival results of a multicentre, randomised, open-label phase 3 study (KEYNOTE-006). Lancet. 2017;390:1853-1862. 10.1016/S0140-6736(17)31601-X 28822576

[33] Weber J, Mandala M, Del Vecchio M, et al. Adjuvant nivolumab versus ipilimumab in resected stage III or IV melanoma. N Engl J Med. 2017;377:1824-1835. 10.1056/NEJMoa1709030 28891423

[34] Foiani G, Melchiotti E, Capello K, et al. PD-L1, PD-1, and CTLA-4 mRNA In situ expression by canine oral melanoma cells and immune cells of the tumour microenvironment. Vet Comp Oncol. 2025;23:141-151. 10.1111/vco.13039 39789732 PMC12082799

[35] He X, Gao Y, Deng Y, et al. The comparative oncology of canine malignant melanoma in targeted therapy: a systematic review of In vitro experiments and animal model reports. Int J Mol Sci. 2024;25:10387. 10.3390/ijms251910387 PMC11476434 39408717

[36] Hernandez B, Adissu HA, Wei BR, Michael H, Merlino G, Simpson R. Naturally occurring canine melanoma as a predictive comparative oncology model for human mucosal and other triple wild-type melanomas. Int J Mol Sci. 2018;19:394. 10.3390/ijms19020394 PMC5855616 29385676

[37] Maekawa N, Konnai S, Nishimura M, et al. PD-L1 immunohistochemistry for canine cancers and clinical benefit of anti-PD-L1 antibody in dogs with pulmonary metastatic oral malignant melanoma. NPJ Precis Oncol. 2021;5:10. 10.1038/s41698-021-00147-6 33580183 PMC7881100

[38] Maekawa N, Konnai S, Okagawa T, et al. Immunohistochemical analysis of PD-L1 expression in canine malignant cancers and PD-1 expression on lymphocytes in canine oral melanoma. PLoS One. 2016;11:e0157176. 10.1371/journal.pone.0157176 27276060 PMC4898770

[39] Prouteau A, Andre C. Canine melanomas as models for human melanomas: clinical, histological, and genetic comparison. Genes (Basel). 2019;10:10. 10.3390/genes10070501 PMC6678806 31262050

[40] de Nardi AB, Dos Santos HR, Fonseca-Alves CE, et al. Diagnosis, prognosis and treatment of canine cutaneous and subcutaneous mast cell tumors. Cells. 2022;11:11. 10.3390/cells11040618 PMC8870669 35203268

[41] Ito D, Frantz AM, Modiano JF. Canine lymphoma as a comparative model for human non-Hodgkin lymphoma: recent progress and applications. Vet Immunol Immunopathol. 2014;159:192-201. 10.1016/j.vetimm.2014.02.016 24642290 PMC4994713

[42] Zandvliet M . Canine lymphoma: a review. Vet Q. 2016;36:76-104. 10.1080/01652176.2016.1152633 26953614

[43] Veterinary cooperative oncology group—common terminology criteria for adverse events (VCOG-CTCAE) following chemotherapy or biological antineoplastic therapy in dogs and cats v1.1. Vet Comp Oncol. 2016;14:417-446. 10.1111/vco.283 28530307

[44] Lynch S, Savary-Bataille K, Leeuw B, Argyle DJ. Development of a questionnaire assessing health-related quality-of-life in dogs and cats with cancer. Vet Comp Oncol. 2011;9:172-182. 10.1111/j.1476-5829.2010.00244.x 21848620

[45] Nguyen SM, Thamm DH, Vail DM, London CA. Response evaluation criteria for solid tumours in dogs (v1.0): a veterinary cooperative oncology group (VCOG) consensus document. Vet Comp Oncol. 2015;13:176-183. 10.1111/vco.12032 23534501

[46] Vail DM, Michels GM, Khanna C, Selting KA, London CA, Veterinary Cooperative Oncology Group. Response evaluation criteria for peripheral nodal lymphoma in dogs (v1.0)—a veterinary cooperative oncology group (VCOG) consensus document. Vet Comp Oncol. 2010;8:28-37. 10.1111/j.1476-5829.2009.00200.x 20230579

[47] Borghaei H, Paz-Ares L, Horn L, et al. Nivolumab versus docetaxel in advanced nonsquamous non-small-cell lung cancer. N Engl J Med. 2015;373:1627-1639. 10.1056/NEJMoa1507643 26412456 PMC5705936

[48] Brahmer J, Reckamp KL, Baas P, et al. Nivolumab versus docetaxel in advanced squamous-cell non-small-cell lung cancer. N Engl J Med. 2015;373:123-135. 10.1056/NEJMoa1504627 26028407 PMC4681400

[49] Garon EB, Rizvi NA, Hui R, et al. Pembrolizumab for the treatment of non-small-cell lung cancer. N Engl J Med. 2015;372:2018-2028. 10.1056/NEJMoa1501824 25891174

[50] Hamanishi J, Mandai M, Ikeda T, et al. Safety and antitumor activity of anti-PD-1 antibody, nivolumab, in patients with platinum-resistant ovarian cancer. J Clin Oncol. 2015;33:4015-4022. 10.1200/JCO.2015.62.3397 26351349

[51] Larkin J, Chiarion-Sileni V, Gonzalez R, et al. Combined nivolumab and ipilimumab or monotherapy in untreated melanoma. N Engl J Med. 2015;373:23-34. 10.1056/NEJMoa1504030 26027431 PMC5698905

[52] Le DT, Uram JN, Wang H, et al. PD-1 blockade in Tumors with mismatch-repair deficiency. N Engl J Med. 2015;372:2509-2520. 10.1056/NEJMoa1500596 26028255 PMC4481136

[53] Topalian SL, Hodi FS, Brahmer JR, et al. Safety, activity, and immune correlates of anti-PD-1 antibody in cancer. N Engl J Med. 2012;366:2443-2454. 10.1056/NEJMoa1200690 22658127 PMC3544539

[54] Gilvetmab [package insert]. Merck Animal Health; 2024. https://merckusa.cvpservice.com/product/basic/view/1047586

[55] Mao Y, Xie H, Lv M, et al. The landscape of objective response rate of anti-PD-1/L1 monotherapy across 31 types of cancer: a system review and novel biomarker investigating. Cancer Immunol Immunother. 2023;72:2483-2498. 10.1007/s00262-023-03441-3 37022474 PMC10992474

[56] Larroquette M, Domblides C, Lefort F, et al. Combining immune checkpoint inhibitors with chemotherapy in advanced solid tumours: a review. Eur J Cancer. 2021;158:47-62. 10.1016/j.ejca.2021.09.013 34655837

[57] Lynch C, Pitroda SP, Weichselbaum RR. Radiotherapy, immunity, and immune checkpoint inhibitors. Lancet Oncol. 2024;25:e352-e362. 10.1016/S1470-2045(24)00075-5 39089313

[58] Takada K, Takamori S, Brunetti L, Crucitti P, Cortellini A. Impact of neoadjuvant immune checkpoint inhibitors on surgery and perioperative complications in patients with non-small-cell lung cancer: a systematic review. Clin Lung Cancer. 2023;24:e585. 10.1016/j.cllc.2023.08.017 37741717

[59] Mao S, Zhou F, Liu Y, et al. ICI plus chemotherapy prolonged survival over ICI alone in patients with previously treated advanced NSCLC. Cancer Immunol Immunother. 2022;71:219-228. 10.1007/s00262-021-02974-9 34097116 PMC10991562

[60] Wang X, Niu X, An N, Sun Y, Chen Z. Comparative efficacy and safety of immunotherapy alone and in combination with chemotherapy for advanced non-small cell lung cancer. Front Oncol. 2021;11:611012. 10.3389/fonc.2021.611012 33816241 PMC8013714

[61] Yu Z, Liang C, Xu Q, et al. The safety and efficacy of neoadjuvant PD-1 inhibitor plus chemotherapy for patients with locally advanced gastric cancer: a systematic review and meta-analysis. Int J Surg. 2024;111:1415-1426. 10.1097/JS9.0000000000002056 PMC11745722 39172720

[62] Li B, Jin J, Guo D, Tao Z, Hu X. Immune checkpoint inhibitors combined with targeted therapy: the recent advances and future potentials. Cancers (Basel). 2023;15:15. 10.3390/cancers15102858 PMC10216018 37345194

[63] Ma X, Zhang Y, Wang S, Wei H, Yu J. Immune checkpoint inhibitor (ICI) combination therapy compared to monotherapy in advanced solid cancer: a systematic review. J Cancer. 2021;12:1318-1333. 10.7150/jca.49174 33531977 PMC7847663

[64] Villacampa G, Cresta Morgado P, Carita L, et al. Safety and efficacy of antibody-drug conjugates plus immunotherapy in solid tumours: a systematic review and meta-analysis. Cancer Treat Rev. 2024;131:102847. 10.1016/j.ctrv.2024.102847 39454548

[65] New listings in AVMA Veterinary Clinical Trials Registry . AVMA News. AVMA Publications: American Veterinary Medical Association; 2024. https://www.avma.org/news/new-listings-avma-veterinary-clinical-trials-registry-september-2024

[66] Deguchi T, Maekawa N, Konnai S, et al. Enhanced systemic antitumour immunity by hypofractionated radiotherapy and anti-PD-L1 therapy in dogs with pulmonary metastatic oral malignant melanoma. Cancers (Basel). 2023;15:15. 10.3390/cancers15113013 PMC10252299 37296981

[67] Villacampa G, Navarro V, Matikas A, et al. Neoadjuvant immune checkpoint inhibitors plus chemotherapy in early breast cancer: a systematic review and meta-analysis. JAMA Oncol. 2024;10:1331-1341. 10.1001/jamaoncol.2024.3456 39207778 PMC12422158

[68] Park HJ, Kim KW, Pyo J, et al. Incidence of pseudoprogression during immune checkpoint inhibitor therapy for solid tumors: a systematic review and meta-analysis. Radiology. 2020;297:87-96. 10.1148/radiol.2020200443 32749204 PMC7526949

[69] Ma Y, Wang Q, Dong Q, Zhan L, Zhang J. How to differentiate pseudoprogression from true progression in cancer patients treated with immunotherapy. Am J Cancer Res. 2019;9:1546-1553. 31497342 PMC6726978

[70] Hodi FS, Hwu WJ, Kefford R, et al. Evaluation of immune-related response criteria and RECIST v1.1 in patients with advanced melanoma treated with Pembrolizumab. J Clin Oncol. 2016;34:1510-1517. 10.1200/JCO.2015.64.0391 26951310 PMC5070547

[71] Hodi FS, Ballinger M, Lyons B, et al. Immune-modified response evaluation criteria in solid tumors (imRECIST): refining guidelines to assess the clinical benefit of cancer immunotherapy. J Clin Oncol. 2018;36:850-858. 10.1200/JCO.2017.75.1644 29341833

[72] Seymour L, Bogaerts J, Perrone A, et al. iRECIST: guidelines for response criteria for use in trials testing immunotherapeutics. Lancet Oncol. 2017;18:e143-e152. 10.1016/S1470-2045(17)30074-8 28271869 PMC5648544

[73] Wolchok JD, Hoos A, O'Day S, et al. Guidelines for the evaluation of immune therapy activity in solid tumors: immune-related response criteria. Clin Cancer Res. 2009;15:7412-7420. 10.1158/1078-0432.CCR-09-1624 19934295

[74] Igase M, Inanaga S, Tani K, et al. Long-term survival of dogs with stage 4 oral malignant melanoma treated with anti-canine PD-1 therapeutic antibody: a follow-up case report. Vet Comp Oncol. 2022;20:901-905. 10.1111/vco.12829 35535636

[75] Jakubovic BD, Vecillas LL, Jimenez-Rodriguez TW, Sanchez-Sanchez S, Castells M. Drug hypersensitivity in the fast lane: what clinicians should know about phenotypes, endotypes, and biomarkers. Ann Allergy Asthma Immunol. 2020;124:566-572. 10.1016/j.anai.2020.04.005 32302769

[76] El Osta B, Hu F, Sadek R, et al. Not all immune-checkpoint inhibitors are created equal: meta-analysis and systematic review of immune-related adverse events in cancer trials. Crit Rev Oncol Hematol. 2017;119:1-12. 10.1016/j.critrevonc.2017.09.002 29065979

[77] Momtaz P, Park V, Panageas KS, et al. Safety of infusing ipilimumab over 30 minutes. J Clin Oncol. 2015;33:3454-3458. 10.1200/JCO.2015.61.0030 26124475 PMC5087314

[78] Park BC, Stone CA Jr, Dewan AK, Johnson DB. Hypersensitivity reactions and immune-related adverse events to immune checkpoint inhibitors: approaches, mechanisms, and models. Immunol Allergy Clin North Am. 2022;42:285-305. 10.1016/j.iac.2021.12.006 35469619

[79] Wang Y, Zhou S, Yang F, et al. Treatment-related adverse events of PD-1 and PD-L1 inhibitors in clinical trials: a systematic review and meta-analysis. JAMA Oncol. 2019;5:1008-1019. 10.1001/jamaoncol.2019.0393 31021376 PMC6487913

[80] Esen BH, Ozbek L, Oguz S, Selcukbiricik F. Characterizing immune checkpoint inhibitor-related cutaneous adverse reactions: a comprehensive analysis of FDA adverse event reporting system (FAERS) database. Heliyon. 2024;10:e33765. 10.1016/j.heliyon.2024.e33765 39071598 PMC11283008

[81] Henderson Berg MH, Del Rincon SV, Miller WH. Potential therapies for immune-related adverse events associated with immune checkpoint inhibition: from monoclonal antibodies to kinase inhibition. J Immunother Cancer. 2022;10:10. 10.1136/jitc-2021-003551 PMC8796266 35086945

[82] Chen TW, Razak AR, Bedard PL, Siu LL, Hansen AR. A systematic review of immune-related adverse event reporting in clinical trials of immune checkpoint inhibitors. Ann Oncol. 2015;26:1824-1829. 10.1093/annonc/mdv182 25888611

[83] Kottschade LA . Incidence and management of immune-related adverse events in patients undergoing treatment with immune checkpoint inhibitors. Curr Oncol Rep. 2018;20:24. 10.1007/s11912-018-0671-4 29511902

[84] Poto R, Troiani T, Criscuolo G, et al. Holistic approach to immune checkpoint inhibitor-related adverse events. Front Immunol. 2022;13:804597. 10.3389/fimmu.2022.804597 35432346 PMC9005797

[85] Postow MA, Sidlow R, Hellmann MD. Immune-related adverse events associated with immune checkpoint blockade. N Engl J Med. 2018;378:158-168. 10.1056/NEJMra1703481 29320654

[86] Yin Q, Wu L, Han L, et al. Immune-related adverse events of immune checkpoint inhibitors: a review. Front Immunol. 2023;14:1167975. 10.3389/fimmu.2023.1167975 37304306 PMC10247998

[87] Igase M, Inanaga S, Nishibori S, et al. Proof-of-concept study of the caninized anti-canine programmed death 1 antibody in dogs with advanced non-oral malignant melanoma solid tumors. J Vet Sci. 2024;25:e15. 10.4142/jvs.23144 38311328 PMC10839171

[88] Hatic H, Sampat D, Goyal G. Immune checkpoint inhibitors in lymphoma: challenges and opportunities. Ann Transl Med. 2021;9:1037. 10.21037/atm-20-6833 34277837 PMC8267255

[89] Armand P, Engert A, Younes A, et al. Nivolumab for relapsed/refractory classic Hodgkin lymphoma after failure of autologous hematopoietic cell transplantation: extended follow-up of the Multicohort Single-Arm Phase II CheckMate 205 trial. J Clin Oncol. 2018;36:1428-1439. 10.1200/JCO.2017.76.0793 29584546 PMC6075855

[90] Seelig DM, Avery AC, Ehrhart EJ, Linden MA. The comparative diagnostic features of canine and human lymphoma. Vet Sci. 2016;3:3. 10.3390/vetsci3020011 28435836 PMC5397114

