# Supplementary material for: Efficacy and safety evaluation of gilvetmab in dogs with melanoma and mast cell tumor
Source: J Vet Intern Med. 2026 Jun 5;40(3):aalag098. doi: 10.1093/jvimsj/aalag098 (PMC13240851; doi:10.1093/jvimsj/aalag098)
Supplement: S1-clean_aalag098 [file s1-clean_aalag098.docx]

Supplementary Methods (S1A, B)

S1A: Serum concentration

The serum concentration of gilvetmab was measured by ELISA. Briefly, 96-well plates were coated overnight at 2-7°C with 0.5ug/well of recombinant canine PD-1 protein. All wells were then washed three times with Phosphate Buffered Saline with Tween 20 (PBST). PD-1-coated wells were blocked with 5% nonfat dry milk in PBST (blocking solution) for 90 minutes at 37oC. The blocking solution was removed and dog sera diluted in blocking solution was added to the PD-1-coated wells and incubated for 1 hour at 37^o^C. Following another washing step, a peroxidase-labeled anti-dog IgG antibody was added to the wells and incubated at 37^o^C for 1 hour. All wells were washed again and a color-generating peroxidase substrate reagent was added to each well. Plates were incubated for 20-40 minutes at 37^o^C and then the optical density of each well was read on a plate reader at a wavelength of 650 nm with a reference wavelength of 450 nm. A standard curve was generated for each plate using a control sample with a known concentration of gilvetmab. Serum concentration of gilvetmab in test samples was determined using a 4-parameter standard curve fit equation calculated using SoftMax® Pro Version 6.3 Software.

S1B: Target engagement

The binding of gilvetmab to canine lymphocyte populations following administration of gilvetmab to dogs was determined by flow cytometry. Briefly, blood was diluted with an equal amount of sterile phosphate buffered saline (PBS). The blood/PBS mixture was layered over the lymphocyte separation medium (LSM) and centrifuged at 400 RCF for 30 minutes at room temperature. The top layer of serum/PBS was removed; the middle PBMC layer was transferred to a new 50 mL conical tube. The tube was filled with PBS and centrifuged at 1,200 RPM for 5 minutes. The supernatant was aspirated. The pellet was resuspended in 500 μL of RPMI COMPLETE and counted. Cells were kept on ice until ready to use. For the binding assay, 100 μL of cells were added at a density of 2.5 x 106 cells/mL to each well of a 96-well round-bottom plate, washed with 150 μL of FACS buffer per well, centrifuged at 1,800 RPM for 2 minutes, and incubated at room temperature for 10 minutes. Then, 50 μL of primary antibodies diluted to 5ug/mL in FACS buffer were directly added to their appropriate wells and incubate for 30 minutes at room temperature. Samples were washed twice; incubated in 50 μL/well of secondary antibody diluted 1:200 in FACS buffer in the dark at room temperature for 20 minutes; washed twice; incubated in 50 μL/well of conjugated antibodies diluted in FACS buffer in the dark at room temperature for 20 minutes; washed twice; resuspended in 50 μL of 4% PFA and incubated for 10 minutes in the dark at room temperature. After 2 more washes, the cells were resuspended in 200 μL FACS buffer and read by flow cytometry. Data from samples was collected on a Gallios Flow Cytometer (Beckman Coulter, Indianapolis, IN) in the Flint Animal Cancer Center at Colorado State University. Flow cytometry gating was determined using an isotype matched irrelevant antibody and samples were analyzed using FlowJo v10 software.
