# Supplementary material for: Efficacy and safety evaluation of gilvetmab in dogs with melanoma and mast cell tumor
Source: J Vet Intern Med. 2026 Jun 5;40(3):aalag098. doi: 10.1093/jvimsj/aalag098 (PMC13240851; doi:10.1093/jvimsj/aalag098)
Supplement: Table_S2-clean_aalag098 [file table_s2-clean_aalag098.docx]

Supplementary Table S2. Summary of concomitant medications for all enrolled dogs with melanoma and MCT.

| **Concomitant medication** | **Count (Percentage)**^a^ |
| --- | --- |
| lidocaine | 32 (62.7%) |
| antipruritics, incl. antihistamines, anesthetics, etc | 25 (49.0%) |
| drugs for peptic ulcer and gastro-oesophageal reflux disease (GORD) | 22 (43.1%) |
| opioids | 21 (41.2%) |
| beta-lactam antibacterials, penicillins | 16 (31.4%) |
| antiemetics and antinausea | 15 (29.4%) |
| antiinflammatory and antirheumatic products, non-steroids | 14 (27.5%) |
| electrolyte solutions | 14 (27.5%) |
| joint supplement | 12 (23.5%) |
| other beta-lactam antibacterials | 10 (19.6%) |
| metronidazole | 9 (17.6%) |
| fluoroquinolones | 9 (17.6%) |
| atipamezole | 9 (17.6%) |
| dexmedetomidine | 9 (17.6%) |
| gabapentin | 9 (17.6%) |
| antibiotics for topical use | 7 (13.7%) |
| anesthetics, general | 7 (13.7%) |

^a^Only the first administration of each medication in any given dog at any time from the first dosing date to study end date, inclusively is included in the count calculation, and only frequency of >10% is listed, in descending order.
