# Supplementary material for: Efficacy and safety evaluation of gilvetmab in dogs with melanoma and mast cell tumor
Source: J Vet Intern Med. 2026 Jun 5;40(3):aalag098. doi: 10.1093/jvimsj/aalag098 (PMC13240851; doi:10.1093/jvimsj/aalag098)
Supplement: Table_S3-clean_aalag098 [file table_s3-clean_aalag098.docx]

Supplementary Table S3. Summary of target tumor location(s) in enrolled dogs with melanoma that experienced an objective response. Underlying bone involvement was not specifically evaluated for each target lesion.

| **Melanoma Target Lesion(s)** | **Initial Sum (mm) of Target Lesion(s)** | **Sum (mm) of Target Lesion(s) at Best Overall Response** | **Best Overall Response** | **Regional LN Included in Best Overall Response?** |
| --- | --- | --- | --- | --- |
| L mandibular LN | 20 | 5 | CR | Yes |
| L mandibular LN | 21 | 8 | CR | Yes |
| R caudal mandibular mass + R mandibular LN | 45+16 = 61 | 16+10 = 26 | PR | Yes |
| R upper lip mass | 42 | 26 | PR | No |
| L front fourth digit mass | 20 | 14 | PR | No |

L, left; LN, lymph node; R, right
